# Spin relaxation: is there anything new under the Sun?

**DOI:** 10.5194/mr-3-27-2022

**Published:** 2022-02-09

**Authors:** Bogdan A. Rodin, Daniel Abergel

**Affiliations:** 1 Laboratoire des biomolécules, LBM, Département de chimie, Ecole normale supérieure, PSL University, Sorbonne Université, CNRS, 75005 Paris, France; 2 International Tomography Center, Siberian Branch of the Russian Academy of Science, Novosibirsk 630090, Russia; 3 Physics Department, Novosibirsk State University, Novosibirsk, 630090, Russia​​​​​​​

## Abstract

Spin relaxation has been at the core of many studies since the early days of nuclear magnetic resonance (NMR) and the underlying theory worked out by its founding fathers. This Bloch–Redfield–Abraham relaxation theory has been recently reinvestigated ([Bibr bib1.bibx5]) in the perspective of Lindblad theory of quantum Markovian master equations in order to account for situations where the widely used semi-classical relaxation theory breaks down. In this article, we review the conventional approach of quantum mechanical theory of NMR relaxation and show that, under the usual assumptions, it is equivalent to the Lindblad formulation. We also comment on the debate on semi-classical versus quantum versions of spectral density functions involved in relaxation.

## 1 Introduction

Relaxation is the process through which a system loses energy to its environment to eventually reach a state of thermal equilibrium. Spin–lattice relaxation has been described as the way spins transfer energy to orientation degrees of freedom. Since the early days of nuclear magnetic resonance (NMR), it has been the subject of numerous studies, both theoretical and encompassing a wide range of domains of applications. NMR relaxation theory really was contemporary in the early days of NMR, and it was formalized by several of its founding fathers [Bibr bib1.bibx8]. This is a rather usual approach to the theory of open systems, which has been widely used in various domains of physics ([Bibr bib1.bibx28]). From this perspective, relaxation is the result of the dynamical coupling of a small ensemble of spins (the system) coupled to a large ensemble of particles, or degrees of freedom (the lattice), that are at thermal (Boltzmann) equilibrium and are endowed with an infinite heat capacity, thereby constituting a thermal reservoir. This very general approach has led to many theoretical predictions with far-reaching practical applications in the domain of magnetic resonance spectroscopy. In particular, the role played by molecular motions has been put to good use to extract dynamical information on complex molecular objects. Thus, with the development of spin engineering techniques, it has become possible to measure selected relaxation rates with high accuracy in a broad range of problems, with the prospect of relating such observables to models of molecular dynamics.

It has been shown recently by [Bibr bib1.bibx5] that the formulation of Redfield's semi-classical theory of relaxation widely used by NMR spectroscopists may lead to erroneous predictions in the case of a two-spin system prepared in high-order states, such as singlet spin states.

Such an unexpected behavior was ascribed to the fact that some of the assumptions of the theory may not be fulfilled in NMR systems, and the authors solved the problem by making use of the Lindblad operator, which is commonly used in the theory of open quantum systems to account for dissipative Markovian phenomena, i.e., relaxation processes. Among other properties, the structure of the Lindblad operator ensures that the fundamental properties of the density operator ρ ( ρ , which is hermitian and definite positive, and Tr(ρ)=1 ) are preserved [Bibr bib1.bibx18]. This article has sparked renewed interest regarding the general theory of relaxation, as first elaborated by Bloch, and its connection with the Lindblad theory of quantum dissipative systems ([Bibr bib1.bibx3]). This approach has been generalized in several recent works [Bibr bib1.bibx20].

The traditional description of NMR relaxation relies on the description of both the spin system and the lattice as quantum systems, an approach that leads to the celebrated Redfield equation ([Bibr bib1.bibx6]; see also [Bibr bib1.bibx13], for a more recent account on spin lattice relaxation). As far as the description of the lattice is concerned, this approach is challenging, and actually untractable, as a quantized description of the degrees of freedom involved in molecular motions (multiple bond rotations, overall tumbling of a molecule, etc.) is not manageable in practice, even for small molecular systems. For this reason, an alternative relaxation theory, where the spins are treated as quantum objects and the lattice is described with classical functions of the lattice degrees of freedom, was developed. This semi-classical approach has far-reaching practical consequences, as spin relaxation can then, in principle, be described using classical models of dynamics for molecular motions, and it has been extensively used over the years to describe and interpret spin relaxation experiments. However, this semi-classical theory predicts a non-Boltzmann equilibrium density operator, which requires an ad hoc thermal correction to the relaxation equations [Bibr bib1.bibx23]. More elaborate attempts have been made to overcome this limitation by modifying the relaxation operator itself and to enforce a Boltzmann equilibrium of the density operator [Bibr bib1.bibx15]. However, in the traditional semi-classical NMR relaxation approach and its later modifications, the spin–lattice interactions are accounted for by a stochastic, fluctuating Hamiltonian. This has important consequences. First, in the conventional Abraham–Redfield approach, the statistical properties of the (classical) lattice are not constrained by a fluctuation–dissipation kind of theorem that would enforce a Boltzmann equilibrium distribution and, therefore, a lattice temperature. Such a constraint is therefore not applied on the spins. Second, a major difference between the quantum and classical theories of spin relaxation is rooted in the non-commutation of quantum bath operators of the spin–lattice coupling Hamiltonian, which confers particular properties to the spin correlation and spectral density functions that are absent in the semi-classical theory. Both aspects, quantum and statistical, are entangled, and the relations between quantum and classical correlation functions will therefore be discussed.

It is the purpose of this paper to re-investigate these old questions in order to trace the roles and the consequences of the various assumptions of the traditional approach to relaxation developed in the early days of NMR.

## 2 Theory of spin relaxation in a thermal bath: a short review

### 2.1 Derivation of the master equation

The derivation follows the lines of [Bibr bib1.bibx1], [Bibr bib1.bibx6], [Bibr bib1.bibx22], and [Bibr bib1.bibx14]. Consider a spin system in its environment. The Hamiltonian of the spin system $H_{\text{S}}$ accounts for the interaction of the spins with the magnetic fields (Zeeman interaction and interaction with a radio frequency (rf) field) and non-dissipative spin–spin interactions (scalar J coupling and dipolar coupling). The dynamics of the lattice (the bath) are described by $H_{\text{B}}$ , and the spin–bath interaction Hamiltonian is $H_{1}$ , as follows:

1 $$H_{\text{T}}=H_{\text{S}}+H_{\text{B}}+H_{1},$$

and the dynamics of the {spin–bath} system are described by the Liouville equation as follows:

2 $$\dot{\rho }(t)=-i[H_{\text{S}}+H_{\text{B}}+H_{1},\rho (t)].$$

Alternatively, it can be written as follows:

3 $$\dot{\rho }(t)=L_{\text{T}}\rho (t),$$

where the operator $L_{\text{T}}=-i[H_{\text{T}},\cdot ]$ is the total Liouvillian. Introducing the density operator in the interaction representation of the density operator $\rho^{*}(t)=e^{-L_{0}t}\rho (t)$ , where $L_{0}=L_{\text{S}}+L_{\text{B}}$ is the unperturbed Liouvillian, one has the following:

4 $$\frac{\text{d}}{\text{d}t}\rho^{*}(t)=-i[H_{1}^{*}(t),\rho^{*}(t)]=L_{1}^{*}(t)\rho^{*}(t).$$

The Liouville equation is integrated to the second order as follows:

5 $$\begin{array}{rl} \rho^{*}(t) & =\rho^{*}(0)+\int_{0}^{t}\text{d}t_{1}L_{1}^{*}(t_{1})\rho^{*}(0)\\ & +\int_{0}^{t}\text{d}t_{1}\int_{0}^{t_{1}}\text{d}t_{2}L_{1}^{*}(t_{1})L_{1}^{*}(t_{2})\rho^{*}(0)+\ldots \end{array}$$

Taking the derivative, one obtains the following:

6 $$\frac{\text{d}}{\text{d}t}\rho^{*}(t)=L_{1}^{*}(t)\rho^{*}(0)+\int_{0}^{t}\text{d}t^{\prime }L_{1}^{*}(t)L_{1}^{*}(t^{\prime })\rho^{*}(0).$$

Finally, by making the change in the variables $\tau =t-t^{\prime }$ , one obtains the master equation for the total density operator as follows:

7 $$\frac{\text{d}}{\text{d}t}\rho^{*}(t)=L_{1}^{*}(t)\rho^{*}(0)+\int_{0}^{t}\text{d}\tau L_{1}^{*}(t)L_{1}^{*}(t-\tau )\rho^{*}(0).$$

The dynamics restricted to the spin system are obtained by eliminating the bath variables. This is achieved by performing a partial trace over the bath degrees of freedom as follows:

8 $$\begin{array}{rl} \sigma (t) & ={\text{tr}}_{\text{B}}\left\{\rho (t)\right\}={\text{tr}}_{\text{B}}\left\{e^{-iH_{\text{T}}t}\rho (0)e^{iH_{\text{T}}t}\right\}\\ & ={\text{tr}}_{\text{B}}\left\{e^{L_{\text{T}}t}\rho (0)\right\}. \end{array}$$

Hence, from Eq. ([Disp-formula Ch1.E6]), the spin density operator in the interaction representation is as follows:

9 $$\sigma^{*}(t)={\text{tr}}_{\text{B}}\rho^{*}(t),$$

which obeys the following:

10 $$\begin{array}{rl} \frac{\text{d}}{\text{d}t}\sigma^{*}(t) & ={\text{tr}}_{\text{B}}\left\{L_{1}^{*}(t_{1})\rho^{*}(0)\right\}\\ & +\int_{0}^{t}\text{d}t^{\prime }{\text{tr}}_{\text{B}}\left\{L_{1}^{*}(t)L_{1}^{*}(t^{\prime })\rho^{*}(0)\right\}. \end{array}$$

Initially, the system and the bath are assumed to be completely decorrelated, as follows:

11 $$\rho (0)=\rho_{\text{B}}^{e}⊗\sigma (0),$$

where the exact $\rho_{\text{B}}^{e}$ denotes the bath density operator in thermal equilibrium. With these assumptions, one has the following:

12 $$\begin{array}{rl} \frac{\text{d}}{\text{d}t}\sigma^{*}(t) & ={\text{tr}}_{\text{B}}\left\{L_{1}^{*}(t_{1})\rho^{*}(0)\right\}\\ & +\int_{0}^{t}\text{d}t^{\prime }{\text{tr}}_{\text{B}}\left\{L_{1}^{*}(t)L_{1}^{*}(t^{\prime })\rho_{\text{B}}^{e}\right\}\sigma^{*}(0). \end{array}$$

In the latter expression, the term ${\text{tr}}_{\text{B}}\left\{L_{1}^{*}(t_{1})\rho^{*}(0)\right\}$ may be assumed to be zero or can be incorporated in the main system Liouvillian $L_{\text{S}}$ as follows [Bibr bib1.bibx1]:

13 $$\frac{\text{d}}{\text{d}t}\sigma^{*}(t)=\int_{0}^{t}\text{d}t^{\prime }{\text{tr}}_{\text{B}}\left\{L_{1}^{*}(t)L_{1}^{*}(t^{\prime })\rho_{\text{B}}^{e}\right\}\sigma^{*}(0).$$

If the spin density operator is assumed to only moderately depart from its initial state, then the following occurs:

14 $$\frac{\sigma (t)-\sigma (0)}{\sigma (0)}\ll 1,$$

where the density operator varies only slightly from its initial state so that $\sigma^{*}(0)$ can be replaced by $\sigma^{*}(t)$ in Eq. ([Disp-formula Ch1.E12]), as follows [Bibr bib1.bibx1]:

15 $$\frac{\text{d}}{\text{d}t}\sigma^{*}(t)=\int_{0}^{t}\text{d}t^{\prime }{\text{tr}}_{\text{B}}\left\{L_{1}^{*}(t)L_{1}^{*}(t^{\prime })\rho_{\text{B}}^{e}\right\}\sigma^{*}(t).$$

The master equation in the Schrödinger representation can be obtained from Eq. ([Disp-formula Ch1.E15]) as follows:

16 $$\begin{array}{rl} & \frac{\text{d}}{\text{d}t}\sigma^{*}(t)\\ & =\frac{\text{d}}{\text{d}t}e^{-L_{\text{S}}t}\sigma (t)=-L_{\text{S}}e^{-L_{\text{S}}t}\sigma (t)+e^{-L_{\text{S}}t}\frac{\text{d}}{\text{d}t}\sigma (t)\\ & =\int_{0}^{t}\text{d}t^{\prime }{\text{tr}}_{\text{B}}\left\{L_{1}^{*}(t)L_{1}^{*}(t^{\prime })\rho_{\text{B}}^{e}\right\}\sigma^{*}(t). \end{array}$$

Therefore, one has the following:

17 $$\begin{array}{rl} \frac{\text{d}}{\text{d}t}\sigma (t) & =L_{\text{S}}\sigma (t)\\ & +e^{L_{\text{S}}t}\int_{0}^{t}\text{d}t^{\prime }{\text{tr}}_{\text{B}}\left\{L_{1}^{*}(t)L_{1}^{*}(t^{\prime })\rho_{\text{B}}^{e}\right\}\sigma^{*}(t). \end{array}$$

Reverting to the Schrödinger representation $\sigma^{*}(t)=e^{-tL_{\text{S}}}\sigma (t)$ , one obtains the following master equation in the Schrödinger representation:

18 $$\frac{\text{d}}{\text{d}t}\sigma (t)=L_{\text{S}}\sigma (t)+\int_{0}^{t}\text{d}t^{\prime }{\text{tr}}_{\text{B}}\left\{L_{1}L_{1}^{*}(t^{\prime }-t)\rho_{\text{B}}^{e}\right\}\sigma (t).$$

A derivation is given in Appendix [App App1.Ch1.S1] for reference. The term ${\text{tr}}_{\text{B}}\left\{L_{1}L_{1}^{*}(t^{\prime }-t)\rho_{\text{B}}^{e}\right\}$ in Eq. ([Disp-formula Ch1.E18]) is a correlation operator acting on the spin system. It projects the system–bath (spin–lattice) coupling onto the bath degrees of freedom. This spin operator therefore carries the statistical properties of the bath, described by its equilibrium and stationary density operator. Finally, assuming that the correlation operator decays to zero in a time $\tau_{c}$ much shorter than the period over which the density matrix varies significantly, the upper limit of the integral can be extended to +∞ . As above, making the change in the variables $\tau =t-t^{\prime }$ , one obtains the following:

19 $$\frac{\text{d}}{\text{d}t}\sigma (t)=L_{\text{S}}\sigma (t)+\int_{0}^{+\infty }\text{d}\tau {\text{tr}}_{\text{B}}\left\{L_{1}L_{1}^{*}(-\tau )\rho_{\text{B}}^{e}\right\}\sigma (t),$$

or, in the following Hamiltonian representation, the following:

20 $$\begin{array}{rl} \frac{\text{d}}{\text{d}t}\sigma (t) & =-i[H_{\text{S}},\sigma (t)]\\ & -\int_{0}^{+\infty }\text{d}\tau {\text{tr}}_{\text{B}}\left\{\left[H_{1},[H_{1}^{*}(-\tau ),\rho_{\text{B}}^{e}\sigma (t)]\right]\right\}. \end{array}$$

One, therefore, obtains a master equation of the Redfield kind, as follows:

21 $$\frac{\text{d}}{\text{d}t}\sigma (t)=-i[H_{\text{S}},\sigma (t)]+R\sigma (t),$$

where $R\;•=-\int_{0}^{+\infty }\text{d}\tau \;{\text{tr}}_{\text{B}}\left\{\left[H_{1},[H_{1}^{*}(-\tau ),\rho_{\text{B}}^{e}\;•]\right]\right\}$ is the Redfield (relaxation) operator.

### 2.2 Formulation of the master equation in operator form

In spin relaxation theory, it is customary to express the relaxation equation in the operator form, which often provides a clearer representation of the spin–bath coupling dynamics. Here, the coupling Hamiltonian is assumed to have the form of a sum of terms, each of which factorizes into a product of lattice $B^{q}$ and spin $S^{q}$ operators.

22 $$H_{1}=\sum_{q}S^{q}B^{q},$$

with the interaction representation as follows:

23Bq(t)=eiHBtBqe-iHBt24Sq(t)=eiHStSqe-iHSt.

Using results of the preceding section (Eqs. [Disp-formula Ch1.E16]–[Disp-formula Ch1.E18]), the Redfield equation becomes the following:

25 $$\begin{array}{rl} {\dot{\sigma }}_{\text{S}}^{*}(t) & =-\sum_{q,q^{\prime }}{\text{tr}}_{\text{B}}\int_{0}^{+\infty }\text{d}t^{\prime }[S^{q}(t)B^{q}(t),\\ & [S^{q^{\prime }}(t^{\prime })B^{q^{\prime }}(t^{\prime }),\rho_{B}^{e}\sigma_{\text{S}}^{*}(t)]]. \end{array}$$

Each term in the sum becomes the following:

26 $$\begin{array}{rl} & {\text{tr}}_{\text{B}}\left[S^{q}(t)B^{q}(t),\left[S^{q^{\prime }}(t^{\prime })B^{q^{\prime }}(t^{\prime }),\rho_{B}^{e}\sigma_{\text{S}}^{*}(t)\right]\right]\\ & \;=\left[S^{q}(t),S^{q^{\prime }}(t^{\prime })\sigma_{\text{S}}^{*}(t)\right]\langle B^{q}(t)B^{q^{\prime }}(t^{\prime })\rangle^{e}\\ & \;+\left[\sigma_{\text{S}}^{*}(t)S^{q^{\prime }}(t^{\prime }),S^{q}(t)\right]\langle B^{q^{\prime }}(t^{\prime })B^{q}(t)\rangle^{e}, \end{array}$$

where the notation, as in the following:

27 $$\langle B^{q}(t)B^{q^{\prime }}(t^{\prime })\rangle^{e}={\text{tr}}_{\text{B}}\left\{B^{q}(t)B^{q^{\prime }}(t^{\prime })\rho_{B}^{e}\right\},$$

has been introduced. The $\langle B^{q}(t)B^{q^{\prime }}(t^{\prime })\rangle^{e}$ are the bath (lattice) correlation functions, and in contrast to Eq. ([Disp-formula Ch1.E19]), these denote usual time correlation functions rather than operators.

28 $$\begin{array}{rl} {\dot{\sigma }}_{\text{S}}^{*}(t) & =-\sum_{q,q^{\prime }}\int_{0}^{+\infty }\text{d}t^{\prime }[S^{q}(t),S^{q^{\prime }}(t^{\prime })\sigma_{\text{S}}^{*}(t)]\langle B^{q}(t)B^{q^{\prime }}(t^{\prime })\rangle^{e}\\ & -\sum_{q,q^{\prime }}\int_{0}^{+\infty }\text{d}t^{\prime }[\sigma_{\text{S}}^{*}(t)S^{q^{\prime }}(t^{\prime }),S^{q}(t)]\langle B^{q^{\prime }}(t^{\prime })B^{q}(t)\rangle^{e}. \end{array}$$

Using the conventional decomposition of the spin operators into a sum of eigenoperators of the Liouvillian $L_{\text{S}}=[H_{\text{S}},•]$ as follows:

29 $$[H_{\text{S}},S_{p}^{q}]=\omega_{p}^{q}S_{p}^{q},$$

one has the following:

30 $$S^{q}(t)=e^{iH_{\text{S}}t}S^{q}e^{-iH_{\text{S}}t}=\sum_{p}S_{p}^{q}e^{i\omega_{p}^{q}t},$$

which one obtains from Eq. ([Disp-formula Ch1.E28]), with the change in the integration variable $\tau =t-t^{\prime }$ as follows:

31 $$\begin{array}{rl} {\dot{\sigma }}_{\text{S}}^{*}(t) & =-\sum_{q,q^{\prime },p,p^{\prime }}e^{i(\omega_{p}^{q}+\omega_{p^{\prime }}^{q^{\prime }})t}\int_{0}^{+\infty }\text{d}\tau \left[S_{p}^{q},S_{p^{\prime }}^{q^{\prime }}\sigma_{\text{S}}^{*}(t)\right]\\ & \;\langle B^{q}(t)B^{q^{\prime }}(t-\tau )\rangle^{e}e^{-i\omega_{p^{\prime }}^{q^{\prime }}\tau }\\ & -\sum_{q,q^{\prime },p,p^{\prime }}e^{i(\omega_{p}^{q}+\omega_{p^{\prime }}^{q^{\prime }})t}\int_{0}^{+\infty }\text{d}\tau \left[\sigma_{\text{S}}^{*}(t)S_{p^{\prime }}^{q^{\prime }},S_{p}^{q}\right]\\ & \;\langle B^{q^{\prime }}(t-\tau )B^{q}(t)\rangle^{e}e^{-i\omega_{p^{\prime }}^{q^{\prime }}\tau }. \end{array}$$

Introducing the secular approximation $\omega_{p}^{q}+\omega_{p^{\prime }}^{q^{\prime }}=0$ , so that $p=p^{\prime },q=-q^{\prime }$ , and renaming the indices reduces to the following:

32 $$\begin{array}{rl} {\dot{\sigma }}_{\text{S}}^{*}(t) & =-\sum_{p,q}\int_{0}^{+\infty }\text{d}\tau \left[S_{p}^{-q},S_{p}^{q}\sigma_{\text{S}}^{*}(t)\right]\\ & \;\langle B^{-q}(t)B^{q}(t-\tau )\rangle^{e}e^{-i\omega_{p}^{q}\tau }\\ & -\sum_{p,q}\int_{0}^{+\infty }\text{d}\tau \left[\sigma_{\text{S}}^{*}(t)S_{p}^{q},S_{p}^{-q}\right]\\ & \;\langle B^{q}(t-\tau )B^{-q}(t)\rangle^{e}e^{-i\omega_{p}^{q}\tau }. \end{array}$$

The assumption that the bath is in a stationary state, $[H_{\text{B}},\rho_{B}^{e},]=0$ , confers some properties to the correlation functions. Thus, the bath correlation functions are also stationary. Indeed, one has the following:

33 $$\begin{array}{rl} & \langle B^{q}(t)B^{-q}(t+\tau )\rangle^{e}\\ & ={\text{tr}}_{\text{B}}\left\{e^{iH_{\text{B}}t}B^{q}e^{-iH_{\text{B}}t}e^{iH_{\text{B}}(t+\tau )}B^{-q}e^{-iH_{\text{B}}(t+\tau )}\rho_{B}^{e}\right\}\\ & ={\text{tr}}_{\text{B}}\left\{e^{iH_{\text{B}}(t-\tau )}B^{q}e^{-iH_{\text{B}}(t-\tau )}e^{iH_{\text{B}}t}B^{-q}e^{-iH_{\text{B}}t}\rho_{B}^{e}\right\}\\ & =\langle B^{q}(t-\tau )B^{-q}(t)\rangle^{e}. \end{array}$$

In addition, because $\text{tr}(AB{)}^{*}=\text{tr}(B^{†}A^{†})$ , it is easy to show the following:

34 $$\langle B^{q}(t-\tau )B^{-q}(t)\rangle^{e*}=\langle B^{q}(t)B^{-q}(t-\tau )\rangle^{e}.$$

Besides, using the property that $B^{-q}=B^{q†}$ , as in the following:

35 $$\begin{array}{rl} & \langle B^{-q}(t)B^{q}(t-\tau )\rangle^{e}\\ & =\frac{1}{L}\sum_{f,f^{\prime }}\langle f|e^{iH_{\text{B}}t}B^{-q}e^{-iH_{\text{B}}t}|f^{\prime }\rangle \\ & \;\langle f^{\prime }|e^{iH_{\text{B}}(t-\tau )}B^{q}e^{-iH_{\text{B}}(t-\tau )}e^{-\beta H_{\text{B}}}|f\rangle \\ & =\frac{1}{L}\sum_{f,f^{\prime }}\langle f|B^{-q}|f^{\prime }\rangle e^{ift}e^{-if^{\prime }t}\langle f^{\prime }|B^{q}|f\rangle \\ & \;e^{if^{\prime }(t-\tau )}e^{-if(t-\tau )}e^{-\beta f}\\ & =\frac{1}{L}\sum_{f,f^{\prime }}\langle f|B^{-q}|f^{\prime }\rangle \langle f^{\prime }|B^{q}|f\rangle e^{-i(f^{\prime }-f)\tau }e^{-\beta f}\\ & =\frac{1}{L}\sum_{f,f^{\prime }}|\langle f^{\prime }|B^{q}|f\rangle {|}^{2}e^{-i(f^{\prime }-f)\tau }e^{-\beta f}, \end{array}$$

where the |f〉 are the eigenstates of the bath Hamiltonian, $H_{\text{B}}$ , and $\beta =\frac{\hbar }{kT}$ , with the convention ℏ=1 . In these equation, the notation $L={\text{tr}}_{\text{B}}e^{\beta H_{\text{B}}}$ was introduced.

## 3 The Redfield equation is equivalent to the Lindblad form of the relaxation equation

It is now straightforward to show that the conventional Bloch–Redfield–Abraham perturbative approach of relaxation is equivalent to the Lindblad formulation of dissipative systems. Indeed, by changing indices in the first term, using the property $\omega_{p}^{-q}=-\omega_{p}^{q}$ , and setting τ→-τ , one has, from Eq. ([Disp-formula Ch1.E32]), the following:

36 $$\begin{array}{rl} {\dot{\sigma }}_{\text{S}}^{*}(t) & =-\sum_{p,q}\int_{-\infty }^{0}\text{d}\tau \left[S_{p}^{q},S_{p}^{-q}\sigma_{\text{S}}^{*}(t)\right]\\ & \;\langle B^{q}(t)B^{-q}(t+\tau )\rangle^{e}e^{-i\omega_{p}^{q}\tau }\\ & -\sum_{p,q}\int_{0}^{+\infty }\text{d}\tau \left[\sigma_{\text{S}}^{*}(t)S_{p}^{q},S_{p}^{-q}\right]\\ & \;\langle B^{q}(t-\tau )B^{-q}(t)\rangle^{e}e^{-i\omega_{p}^{q}\tau }. \end{array}$$

Using the stationarity property (Eq. [Disp-formula Ch1.E33]) of the bath correlation functions leads to following:

37σ˙S∗(t)=-∑p,q∫-∞0dτSpq,Sp-qσS∗(t)〈Bq(t-τ)B-q(t)〉ee-iωpqτ-∑p,q∫0+∞dτσS∗(t)Spq,Sp-q〈Bq(t-τ)B-q(t)〉ee-iωpqτ38σ˙S∗(t)≈-12∑p,q∫-∞+∞dτSpq,Sp-qσS∗(t)〈Bq(t-τ)B-q(t)〉ee-iωpqτ-12∑p,q∫-∞+∞dτσS∗(t)Spq,Sp-q〈Bq(t-τ)B-q(t)〉ee-iωpqτ,

so that, in the following:

39 $$\begin{array}{rl} {\dot{\sigma }}_{\text{S}}^{*}(t) & \approx -\frac{1}{2}\sum_{p,q}\left[S_{p}^{q},S_{p}^{-q}\sigma_{\text{S}}^{*}(t)\right]J_{\text{R}}^{q,-q}(\omega_{p}^{q})\\ & -\frac{1}{2}\sum_{p,q}\left[\sigma_{\text{S}}^{*}(t)S_{p}^{q},S_{p}^{-q}\right]J_{\text{R}}^{q,-q}(\omega_{p}^{q})\\ & =\sum_{p,q}J_{\text{R}}^{q,-q}(\omega_{p}^{q})(S_{p}^{-q}\sigma_{\text{S}}^{*}(t)S_{p}^{q}\\ & -\frac{1}{2}\left\{S_{p}^{q}S_{p}^{-q},\sigma_{\text{S}}^{*}(t)\right\}), \end{array}$$

where the right spectral density $J_{\text{R}}^{q,-q}(\omega_{p}^{q})$ of the bath is given by the following:

40 $$\begin{array}{rl} J_{\text{R}}^{q,-q}(\omega_{p}^{q}) & =\int_{-\infty }^{+\infty }\text{d}\tau \langle B^{q}B^{-q}(\tau )\rangle e^{-i\omega_{p}^{q}\tau }\\ & =\int_{-\infty }^{+\infty }\text{d}\tau C_{\text{R}}^{q,-q}(\tau )e^{-i\omega_{p}^{q}\tau }, \end{array}$$

where {⋅,⋅} denotes the anti-commutator. The operator appearing on the right-hand side of Eq. ([Disp-formula Ch1.E39]) is the Lindblad dissipation super-operator, as follows:

41 $$\hat{LD}[A,B]=A•B-\frac{1}{2}\left(BA•+•BA\right),$$

so that Eq. ([Disp-formula Ch1.E39]) becomes the following:

42 $${\dot{\sigma }}_{\text{S}}^{*}(t)=\sum_{p,q}J_{R}^{q,-q}(\omega_{p}^{q})\hat{LD}\left[S_{p}^{-q},S_{p}^{q}\right]\sigma_{\text{S}}^{*}(t).$$

Equation ([Disp-formula Ch1.E42]) (and Eq. [Disp-formula Ch1.E39]) is a Lindblad equation [Bibr bib1.bibx18], which is thus derived from the usual quantized theory of relaxation [Bibr bib1.bibx7].

The fact that this derivation leads to the Lindblad equation is not obvious. In principle, one should not expect the perturbative approach to yield an irreversible dissipative operator equivalent to a Lindblad operator. In fact, this equivalence requires the Markovian and the short correlation time assumptions that make the evolution equation depend on the density operator at the present time t only and not in its previous history. Moreover, it also requires the secular approximation that eliminates the time dependence of the spin operators of the coupling Hamiltonian in Eq. ([Disp-formula Ch1.E28]), leading to Eq. ([Disp-formula Ch1.E32]). The combination of these conditions lead to the semi-group property and the Lindblad form of the relaxation operator.

Another point is worth mentioning. The properties of the correlation functions that emerge through this procedure reflect the properties of the bath operators of the spin–bath coupling Hamiltonian and, therefore, convey additional properties that are not implied by the structure of the Lindblad in Eqs. ([Disp-formula Ch1.E39]) and ([Disp-formula Ch1.E42]). Such properties, arising from the non-commutation of the bath operators and the fact that the lattice is always in a stationary Boltzmann equilibrium (detailed in Sect. [Sec Ch1.S3.SS1] below and in Appendix [App App1.Ch1.S2]), ensure a detailed balance and that the stationary state of the spin system is compatible with the Boltzmann equilibrium of the lattice, that is, the lattice temperature. In other words, the perturbative approach leads to a master equation of the Lindblad form, with additional physical properties that bear constraints from the lattice.

### 3.1 An alternative formulation (equivalent to Lindblad)

It is possible to obtain an alternative and completely equivalent form of the Redfield equation. Expanding Eq. ([Disp-formula Ch1.E32]), one obtains the following:

43 $$\begin{array}{rl} {\dot{\sigma }}_{\text{S}}^{*}(t) & =-\sum_{q}\int_{0}^{\infty }\text{d}\tau S_{p}^{-q}S_{p}^{q}\sigma_{\text{S}}^{*}(t)\langle B^{-q}(\tau )B^{q}\rangle e^{-i\omega_{p}^{q}\tau }\\ & +\sum_{q}\int_{0}^{\infty }\text{d}\tau S_{p}^{-q}\sigma_{\text{S}}^{*}(t)S_{p}^{q}\langle B^{q}B^{-q}(\tau )\rangle e^{-i\omega_{p}^{q}\tau }\\ & +\sum_{q}\int_{0}^{\infty }\text{d}\tau S_{p}^{q}\sigma_{\text{S}}^{*}(t)S_{p}^{-q}\langle B^{-q}(\tau )B^{q}\rangle e^{-i\omega_{p}^{q}\tau }\\ & -\sum_{q}\int_{0}^{\infty }\text{d}\tau \sigma_{\text{S}}^{*}(t)S_{p}^{q}S_{p}^{-q}\langle B^{q}B^{-q}(\tau )\rangle e^{-i\omega_{p}^{q}\tau }. \end{array}$$

As above, the left-hand side spectral density function is defined as follows:

44 $$\begin{array}{rl} J_{\text{L}}^{-q,q}(\omega ) & =\int_{-\infty }^{\infty }\text{d}\tau \langle B^{-q}(\tau )B^{q}\rangle e^{-i\omega \tau }\\ & =\int_{-\infty }^{\infty }\text{d}\tau C_{\text{L}}^{-q,q}(\tau )e^{-i\omega \tau }=e^{-\beta \omega }J_{\text{R}}^{q,-q}(\omega ), \end{array}$$

where β=ℏ/kT ( ℏ=1 ). The latter equations express a Kubo kind of relation ([Bibr bib1.bibx16]), where $J_{\text{L}}^{-q,q}(\omega )=e^{-\beta \omega }J_{\text{R}}^{q,-q}(\omega )$ . A proof thereof is given in the Appendix (see Eqs. [Disp-formula App1.Ch1.S2.E83] and [Disp-formula App1.Ch1.S2.E85]). After some straightforward manipulations, one obtains the following:

45 $$\begin{array}{rl} {\dot{\sigma }}_{\text{S}}^{*}(t) & =-\frac{1}{2}\sum_{q}S_{p}^{-q}S_{p}^{q}\sigma_{\text{S}}^{*}(t)e^{-\beta \omega_{p}^{q}}J_{\text{R}}^{q,-q}(\omega_{p}^{q})\\ & +\frac{1}{2}\sum_{q}S_{p}^{-q}\sigma_{\text{S}}^{*}(t)S_{p}^{q}J_{\text{R}}^{q,-q}(\omega_{p}^{q})\\ & +\frac{1}{2}\sum_{q}S_{p}^{q}\sigma_{\text{S}}^{*}(t)S_{p}^{-q}e^{-\beta \omega_{p}^{q}}J_{\text{R}}^{q,-q}(\omega_{p}^{q})\\ & -\frac{1}{2}\sum_{q}\sigma_{\text{S}}^{*}(t)S_{p}^{q}S_{p}^{-q}J_{\text{R}}^{q,-q}(\omega_{p}^{q}). \end{array}$$

Thus, by collecting and rearranging terms, one obtains the following:

46 $$\begin{array}{rl} {\dot{\sigma }}_{\text{S}}^{*}(t) & =\frac{1}{2}\sum_{q}J_{\text{R}}^{q,-q}(\omega_{p}^{q})\left(\left[S_{p}^{-q},\sigma_{\text{S}}^{*}(t)S_{p}^{q}\right]\right.\\ & \left.-\left[S_{p}^{-q},S_{p}^{q}\sigma_{\text{S}}^{*}(t)\right]e^{-\beta \omega_{p}^{q}}\right). \end{array}$$

The Boltzmann factor can be expanded in a series, as follows:

47 $$\begin{array}{rl} {\dot{\sigma }}_{\text{S}}^{*}(t) & =-\frac{1}{2}\sum_{q}J_{\text{R}}^{q,-q}(\omega_{p}^{q})\left[S_{p}^{-q},\left[S_{p}^{q},\sigma_{\text{S}}^{*}(t)\right]\right]\\ & -\frac{1}{2}\sum_{q}J_{\text{R}}^{q,-q}(\omega_{p}^{q})\\ & \;\left[S_{p}^{-q},S_{p}^{q}\sigma_{\text{S}}^{*}(t)\sum_{n=1}^{\infty }\frac{1}{n!}(-\beta \omega_{p}^{q}{)}^{n}\right]. \end{array}$$

The first term on the right-hand side of this equation is the usual double commutator and the symmetry, while the second term represents the thermal effect of the Boltzmann equilibrium of the lattice. This term, equal to $1-\mathrm{exp}(-\beta \omega_{p}^{q})$ , vanishes for infinite temperature.

## 4 A pseudo-classical version of the Redfield equation

A semi-classical version of the master equation can be extremely useful, allowing one to make use of models derived from the framework of classical mechanics to calculate the spectral density functions. In order to obtain such a theory associated to Eqs. ([Disp-formula Ch1.E39]) and ([Disp-formula Ch1.E42]), additional adjustments are necessary. Indeed, because the $B^{-q}(\tau )$ operators do not commute, the correlation functions of the type $\langle B^{-q}(\tau )B^{q}\rangle$ do not obey the general symmetry rules of classical correlation functions. However, symmetrized correlation functions do commute, and so these symmetrized (quantum mechanical) correlation functions should be introduced in order to obtain a semi-classical theory (which we call pseudo-classical to distinguish it from the theory where the effect of the bath is taken into account only through random functions). A general definition of the classical correlation function of two dynamical variables A and B is as follows ([Bibr bib1.bibx11]):

48 $$C_{AB}(t)=\langle A(t)B^{*}\rangle ,$$

where the brackets indicate classical ensemble average. In the case of stationary processes, the following properties of a correlation function can be deduced. Its complex conjugate $C_{AB}^{*}(t)$ is, therefore, as follows ([Bibr bib1.bibx11]):

49 $$\begin{array}{rl} C_{AB}^{*}(t) & =\langle A(t)B^{*}\rangle^{*}=\langle A^{*}(t)B\rangle =\langle A^{*}B(-t)\rangle \\ & =C_{BA}(-t). \end{array}$$

For an autocorrelation function of A and $C_{AA}(t)$ , one has the following:

50 $$C_{AA}(t{)}^{*}=C_{AA}(-t).$$

Equation ([Disp-formula Ch1.E50]) shows that, in the general case where the autocorrelation function is complex, $C_{AA}(t)=C_{AA}^{r}(t)+iC_{AA}^{i}(t)$ , with CAAr(t)=Re(CAA(t)) and CAAi(t)=Im(CAA(t)) are even and odd functions of time, since $C_{AA}^{*}(t)=C_{AA}^{r}(t)-iC_{AA}^{i}(t)=C_{AA}^{r}(-t)+iC_{AA}^{i}(-t)$ . This implies that the associated spectral density, $J(\omega )=\int_{-\infty }^{+\infty }C_{AA}(t)e^{-i\omega t}\text{d}t$ is real and $J(\omega )=J_{AA}^{e}(\omega )+J_{AA}^{o}(\omega )$ , $J_{AA}^{e}(\omega )=\int_{-\infty }^{+\infty }C_{AA}^{r}(t)e^{-i\omega t}\text{d}t$ , and $J_{AA}^{o}(\omega )=i\int_{-\infty }^{+\infty }C_{AA}^{i}(t)e^{-i\omega t}\text{d}t$ are real and, respectively, even and odd functions.

A semi-classical relaxation theory should provide spectral density functions obeying the general classical mechanics requirements detailed above. It is clear, however, that in the quantum case, where A and B are in general non-commuting operators, the above symmetry relations do no apply. It is nevertheless possible to define a symmetrized correlation function as follows:

51 $$C_{AB}(t)=\frac{1}{2}\left\{\langle A^{†}B(t)\rangle +\langle B^{†}A(-t)\rangle \right\},$$

which is real when $C_{AB}(t)$ is stationary. Note also that the bath operator correlation functions have the following property:

52 $$\begin{array}{rl} \langle B^{q}B^{q^{\prime }}(\tau )\rangle^{*} & =\text{tr}\{B^{q}e^{iH_{\text{B}}\tau }B^{q^{\prime }}e^{-iH_{\text{B}}\tau }\rho^{e}{\}}^{*}\\ & =\text{tr}\{\rho^{e}e^{iH_{\text{B}}\tau }B^{q^{\prime }†}e^{-iH_{\text{B}}\tau }B^{q†}\}\\ & =\langle B^{q^{\prime }†}(\tau )B^{q†}\rangle =\langle B^{q^{\prime }†}B^{q†}(-\tau )\rangle , \end{array}$$

where correlation functions are assumed stationary, so that, in the following:

53〈BqB-q(τ)〉∗=〈Bq(τ)B-q〉=〈BqB-q(-τ)〉54〈B-qBq(τ)〉∗=〈B-q(τ)Bq〉=〈B-qBq(-τ)〉.

Using the definitions $C_{\text{L}}^{-q,q}(\tau )=\langle B^{-q}(\tau )B^{q}\rangle$ and $C_{\text{R}}^{q,-q}(\tau )=\langle B^{q}B^{-q}(\tau )\rangle$ , the relations in Eqs. ([Disp-formula Ch1.E53]) and ([Disp-formula Ch1.E54]) show that the average $C^{q}(\tau )=\frac{1}{2}(C_{\text{L}}^{-q,q}(\tau )+C_{\text{R}}^{q,-q}(\tau ))$ obey the classical correlation function property $C^{q*}(\tau )=C^{q}(-\tau )$ .

A semi-classical version of the Redfield equation is thus obtained by using the spectral density function $J^{q}(\omega )$ obtained from the Fourier transform of the symmetrized correlation function $C^{q}(\tau )$ , as follows:

55 $$\begin{array}{rl} J^{q}(\omega ) & =\frac{1}{2}\left(J_{\text{R}}^{q,-q}(\omega )+J_{\text{L}}^{-q,q}(\omega )\right)\\ & =\frac{1}{2}\left(J_{\text{R}}^{q,-q}(\omega )+e^{-\beta \omega }J_{\text{R}}^{q,-q}(\omega )\right)\\ & =\frac{1+e^{-\beta \omega }}{2}J_{\text{R}}^{q,-q}(\omega ). \end{array}$$

Using Eqs. ([Disp-formula Ch1.E55]) and ([Disp-formula Ch1.E45]), it is therefore possible to derive an alternative expression of the master equation. This gives the following:

56 $$\begin{array}{rl} {\dot{\sigma }}_{\text{S}}^{*}(t) & =-\sum_{q}S_{p}^{-q}S_{p}^{q}\sigma_{\text{S}}^{*}(t)\frac{e^{-\beta \omega_{p}^{q}}}{1+e^{-\beta \omega_{p}^{q}}}J^{q}(\omega_{p}^{q})\\ & +\sum_{q}S_{p}^{-q}\sigma_{\text{S}}^{*}(t)S_{p}^{q}\frac{1}{1+e^{-\beta \omega_{p}^{q}}}J^{q}(\omega_{p}^{q})\\ & +\sum_{q}S^{q}\sigma_{\text{S}}^{*}(t)S_{p}^{-q}\frac{e^{-\beta \omega_{p}^{q}}}{1+e^{-\beta \omega_{p}^{q}}}J^{q}(\omega_{p}^{q})\\ & -\sum_{q}\sigma_{\text{S}}^{*}(t)S_{p}^{q}S_{p}^{-q}\frac{1}{1+e^{-\beta \omega_{p}^{q}}}J^{q}(\omega_{p}^{q}), \end{array}$$

or the following:

57 $$\begin{array}{rl} {\dot{\sigma }}_{\text{S}}^{*}(t) & =-\sum_{q}S_{p}^{-q}S_{p}^{q}\sigma_{\text{S}}^{*}(t)\frac{1}{1+e^{\beta \omega_{p}^{q}}}J^{q}(\omega_{p}^{q})\\ & +\sum_{q}S_{p}^{-q}\sigma_{\text{S}}^{*}(t)S_{p}^{q}\frac{1}{1+e^{-\beta \omega_{p}^{q}}}J^{q}(\omega_{p}^{q})\\ & +\sum_{q}S_{p}^{q}\sigma_{\text{S}}^{*}(t)S_{p}^{-q}\frac{1}{1+e^{\beta \omega_{p}^{q}}}J^{q}(\omega_{p}^{q})\\ & -\sum_{q}\sigma_{\text{S}}^{*}(t)S_{p}^{q}S_{p}^{-q}\frac{1}{1+e^{-\beta \omega_{p}^{q}}}J^{q}(\omega_{p}^{q}). \end{array}$$

Finally, from collecting and rearranging terms, one obtains the following:

58 $$\begin{array}{rl} {\dot{\sigma }}_{\text{S}}^{*}(t) & =\frac{1}{2}\sum_{q}J^{q}(\omega_{p}^{q})\left(\left[S_{p}^{-q},\sigma_{\text{S}}^{*}(t)S_{p}^{q}\right]\frac{1}{1+e^{-\beta \omega_{p}^{q}}}\right.\\ & \left.-\left[S_{p}^{-q},S_{p}^{q}\sigma_{\text{S}}^{*}(t)\right]\frac{1}{1+e^{\beta \omega_{p}^{q}}}\right). \end{array}$$

In view of clarifying the connection between the derivation of the quantum mechanical master equation to further semi-classical approximations, it is interesting to rewrite Eq. ([Disp-formula Ch1.E58]) by expanding the temperature function in terms of the parameters $\beta \omega_{p}^{q}$ , as follows:

59 $$\begin{array}{rl} {\dot{\sigma }}_{\text{S}}^{*}(t) & =\frac{1}{2}\sum_{q}J^{q}(\omega_{p}^{q})\\ & \;\left(\left[S_{p}^{-q},\sigma_{\text{S}}^{*}(t)S_{p}^{q}\right]\left(\frac{1}{2}+\frac{\beta \omega_{p}^{q}}{4}-\frac{(\beta \omega_{p}^{q}{)}^{3}}{48}\right)\right.\\ & \left.-\left[S_{p}^{-q},S_{p}^{q}\sigma_{\text{S}}^{*}(t)\right]\left(\frac{1}{2}-\frac{\beta \omega_{p}^{q}}{4}+\frac{(\beta \omega_{p}^{q}{)}^{3}}{48}\right)\right)\\ & =\frac{1}{4}\sum_{q}J^{q}(\omega_{p}^{q})\left[S_{p}^{-q},\left[S_{p}^{q},\sigma_{\text{S}}^{*}(t)\right]\right]\\ & +\frac{1}{2}\sum_{q}J^{q}(\omega_{p}^{q})\left(\frac{\beta \omega_{p}^{q}}{4}-\frac{(\beta \omega_{p}^{q}{)}^{3}}{48}+\cdots \right)\\ & \;\left[S_{p}^{-q},\left\{S_{p}^{q},\sigma_{\text{S}}^{*}(t)\right\}\right]. \end{array}$$

Equation ([Disp-formula Ch1.E59]) contains a double commutator term weighted by the spectral densities $J^{q}(\omega_{p}^{q})$ , which are constructed so as to obey the general symmetry properties of classical spectral density functions (see Eq. [Disp-formula Ch1.E48] and below) and are therefore adapted to a semi-classical version of the relaxation master equation. Then, the $J^{q}(\omega )$ are obtained from classical lattice functions of a fluctuating Hamiltonian, whilst the semi-classical master equation obeys detailed balance. The second term of Eq. ([Disp-formula Ch1.E59]) introduces a lattice-temperature-dependent contribution. However, this term vanishes when the bath operators of the spin–bath coupling Hamiltonian commute, $[B^{-q},B^{q}]=0$ . According to Eq. ([Disp-formula App1.Ch1.S2.E85]), the latter condition also implies that one has the equality of $J_{\text{L}}^{-q,q}(\omega )=J_{\text{R}}^{q,-q}(\omega )$ , meaning that the lattice temperature is infinite. Stated otherwise, this means that a finite lattice temperature is incompatible with commuting bath operators. However, in general, $[B^{-q},B^{q}]\neq 0$ , so that a detailed balance assumption, or property, which is ensured by the model of a bath in thermal Boltzmann equilibrium, is conveyed to the spin system through the non-commutation of the bath operators $B^{q}$ . Each term in the series expansion on the right-hand side of Eq. ([Disp-formula Ch1.E59]) explicitly gives the effect of non-commutation at each order of the parameter $\beta \omega_{p}^{q}$ . The first-order approximation provides the adequate expression in the high temperature limit (see below).

The final relaxation super-operator, which defines the relaxation of the density matrix as ${\dot{\sigma }}_{\text{S}}^{*}(t)=\hat{\Gamma }\sigma^{*}(t)$ , may be written as follows:

60 $$\hat{\Gamma }=-\frac{1}{4}\sum_{q,p}J^{q}(\omega_{q}^{p}){\hat{D}}^{\beta }(\omega_{p}^{q})\left[S_{p}^{-q},S_{p}^{q}\right].$$

Here, ${\hat{D}}^{\beta }$ is the thermalized double commutator super-operator that composes the super-operator from the two operators A,B in the following way:

61 $${\hat{D}}^{\beta }(\omega )\left[A,B\right]=\left[A,f_{\beta }(\omega )B•-•Bf_{\beta }(-\omega )\right],$$

where $f_{\beta }(\omega )=\frac{2}{1+e^{\beta \omega }}$ , and the dot is the place for the operator on which super-operator is applied. It is easy to see that when T→∞ , then $f_{\beta }(\omega )\to 1$ , and the ${\hat{D}}^{\beta }(\omega )$ becomes a double commutator super-operator, and Eq. ([Disp-formula Ch1.E60]) becomes a standard sum of the double commutator super-operators. This equation is completely equivalent to Eqs. ([Disp-formula Ch1.E42]) and ([Disp-formula Ch1.E47]). Nevertheless, the form of the Lindblad dissipator is still easily recognizable, as one may substitute Eq. ([Disp-formula Ch1.E55]) into Eq. ([Disp-formula Ch1.E42]) and obtain the following:

$${\dot{\sigma }}_{\text{S}}^{*}(t)=\sum_{p,q}f_{\beta }(\omega_{p}^{q})J^{q,-q}(\omega_{p}^{q})\hat{LD}\left[S_{p}^{-q},S_{p}^{q}\right]\sigma_{\text{S}}^{*}(t).$$

Equation ([Disp-formula Ch1.E58]) partially decouples the statistical and dynamical properties of the heat reservoir. Statistical properties, unrelated to the dynamics, which are functions of the temperature, are contained in the temperature factors, whereas the information about quantum mechanical bath dynamics is contained in the Fourier transform of the symmetrized (here $\frac{1}{2}(J_{R}^{q,-q}+J_{L}^{-q,q})$ ) correlation functions. However, the latter still implicitly depend on the temperature through the trace over the bath degrees of freedom.

### The case of real correlation functions

The above form of the relaxation master equation (Eqs. [Disp-formula Ch1.E58]–[Disp-formula Ch1.E60]) is suitable for semi-classical approximations of relaxation where classical correlation functions can be used instead of quantum ones that are, in general, impossible to calculate or compute. It is often the case that the classical correlation functions, calculated from classical models of dynamics, such as diffusion, jumps, etc., are real functions of time. The condition of Eq. ([Disp-formula Ch1.E50]) then implies that the spectral density function $J^{q}(\omega )$ is even $J^{q}(-\omega )=J^{q}(\omega )$ .

## 5 Simplifications in the high temperature approximation

When the largest eigenvalue of the operators $S_{p}^{q}$ is such that $max(\beta \omega_{p}^{q})\ll 1$ , then Eq. ([Disp-formula Ch1.E58]) takes a simpler form, as follows:

62 $$\begin{array}{rl} {\dot{\sigma }}_{\text{S}}^{*}(t) & =\frac{1}{4}\sum_{q}-J^{q}(\omega_{p}^{q})\left[S_{p}^{-q},\left[S_{p}^{q},\sigma (t)\right]\right]\\ & +\frac{\beta \omega_{p}^{q}}{2}J^{q}(\omega_{p}^{q})\left[S_{p}^{-q},\left\{S_{p}^{q},\sigma (t)\right\}\right] \end{array}$$

where {.,.} denotes the anti-commutator.

### 5.1 The low-order approximation

The assumption that the density operator is always close to the fully disordered state, $||\sigma -\frac{1}{A}||\ll 1$ , where A is the dimension of the density operator, was made by [Bibr bib1.bibx22]. Limiting the expansion to the zeroth order, Eq. ([Disp-formula Ch1.E62]) becomes the following ([Bibr bib1.bibx14]):

63 $$\begin{array}{rl} {\dot{\sigma }}_{\text{S}}^{*}(t) & =\frac{1}{4}\sum_{q}-J^{q}(\omega_{p}^{q})\left[S_{p}^{-q},\left[S_{p}^{q},\sigma (t)\right]\right]\\ & +\frac{\beta \omega_{p}^{q}}{A}J^{q}(\omega_{p}^{q})\left[S_{p}^{-q},S_{p}^{q}\right]. \end{array}$$

Moreover, using the property, Eq. ([Disp-formula Ch1.E29]), and the Taylor expansion of the exponential, it is straightforward to show the following:

64 $$e^{-\beta H_{\text{S}}}S_{p}^{q}e^{\beta H_{\text{S}}}=e^{-\beta \omega_{p}^{q}}S_{p}^{q}.$$

Therefore, when the density operator is in the thermal equilibrium determined by the Hamiltonian $H_{\text{S}}$ , $\sigma^{\mathrm{eq}}={\text{tr}}_{\text{B}}({\mathrm{exp}}^{-\beta H_{\text{S}}}{)}^{-1}{\mathrm{exp}}^{-\beta H_{\text{S}}}$ , one can show that $R\sigma^{\mathrm{eq}}=0$ , where R is defined by Eq. ([Disp-formula Ch1.E58]). Then, discarding terms that are second order or higher in $max(\beta \omega_{p}^{q})\ll 1$ , in Eq. ([Disp-formula Ch1.E62]), one obtains the semi-classical formulation of the Redfield equation as follows ([Bibr bib1.bibx1]):

65 $$\begin{array}{rl} & \frac{\text{d}}{\text{d}t}(\sigma_{\text{S}}^{*}(t)-\sigma^{\mathrm{eq}})=\\ & \;-\frac{1}{2}\sum_{q,p}J^{q}(\omega_{p}^{q})\left[S_{p}^{-q},\left[S_{p}^{q},\sigma (t)-\sigma^{\mathrm{eq}}\right]\right]. \end{array}$$

The evolution of the expected value of an operator is given by the alternative master equation as follows:

66 $$\begin{array}{rl} \frac{\text{d}}{\text{d}t}\langle O\rangle & =\frac{1}{4}\sum_{q}-J^{q}(\omega_{p}^{q})\langle \left[S_{p}^{q},\left[S_{p}^{-q},O\right]\right]\rangle \\ & +\beta \omega_{p}^{q}\sum_{q}J^{q}(\omega_{p}^{q})\langle \left\{S_{p}^{q},\left[O,S_{p}^{-q}\right]\right\}\rangle . \end{array}$$

In this expression, the second term on the right-hand side contains the thermal contributions to relaxation and can be selectively neglected for terms that are higher than the first order in $\beta \omega_{p}^{q}$ . That is, each term in the development is such that, in the following:

67 $$\begin{array}{rl} & \text{tr}\left(\left\{S_{p}^{q},\left[O,S_{p}^{-q}\right]\right\}\sigma (t)\right)\\ & \;\ll min\left(\text{tr}\left(\left[S_{p}^{q},\left[S_{p}^{-q},O\right]\right]\sigma (t)\right)\right), \end{array}$$

which, at all times, can be discarded. Eq. ([Disp-formula Ch1.E67]) is, in principle, a less stringent condition and may provide criteria for the quasi- or pseudo-classical approximation, which is a test that can be verified a posteriori. It may, thus, provide a way to select which parts of the density operator can be discarded (neglected) and which must be retained in order to obtain an approximate analytical solution.

### 5.2 The simple case of a two-spin system

#### 5.2.1 Double commutator versus thermal contributions

The differences in the contributions between the first (double commutator) and the second (thermal) series of terms in Eq. ([Disp-formula Ch1.E66]) are illustrated in Figs. [Fig Ch1.F1] and [Fig Ch1.F2] in the case of a pair of like spins $\frac{1}{2}$ subject to the relaxation caused by a mutual dipolar (dipole–dipole – DD) interaction and the presence of a randomly fluctuating field (ran). Simulations were performed, assuming Lorentzian spectral density functions, as follows:

68 $$J_{\text{DD, ran}}^{\text{cl}}(\omega )=\frac{2\tau_{\text{C},\text{ran}}}{1+\omega^{2}\tau_{\text{C, ran}}^{2}},$$

with correlation times $\tau_{\text{ran}}=60$ ps and $\tau_{\text{C}}=8$ ps for the random field and dipolar interactions, respectively. The dipolar coupling constant $b_{12}=-(\mu_{0}/4\pi )\gamma_{I}^{2}\hbar r_{12}^{-3}$ refers to the dipole–dipole coupling constant, where $\gamma_{I}$ is the gyromagnetic ratio, and $r_{12}$ is the internuclear distance. In these simulations, $b_{12}=35\times 10^{3}$ Hz. These values were chosen to give $T_{1}\approx 2$ s and $T_{\text{S}}\approx 20$ s.

**Figure 1 Ch1.F1:**
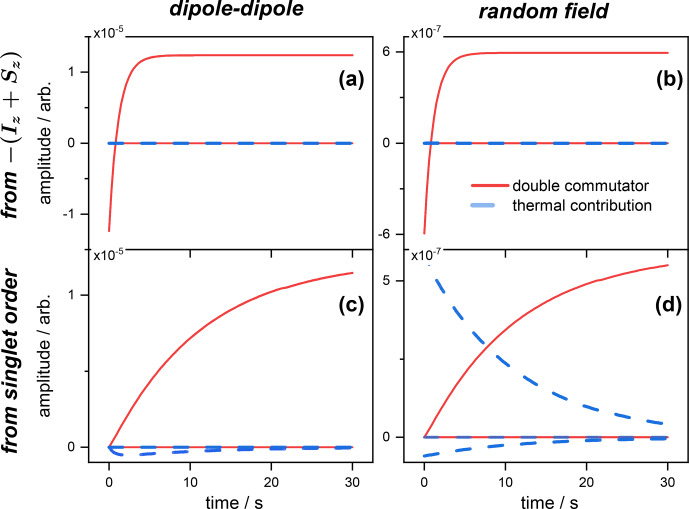
Expected values of the magnetization spin operator $O=I_{z}+S_{z}$ , with the contributions of the double commutator (red curves) and thermal (blue curves) parts of the Redfield relaxation operator to the magnetization from the different operators $S_{p}^{q}$ (see Table [Table App1.Ch1.S3.T1]). Panels **(a)** and **(b)** correspond, respectively, to the dipolar and random field relaxation of the spins inverted from a Boltzmann equilibrium. Panels **(c)** and **(d)** correspond, respectively, to the dipolar and random field relaxation of the spins initially prepared in a singlet state. Simulations were performed using the Scilab software (https://scilab.org, last access: 31 January 2022).

**Figure 2 Ch1.F2:**
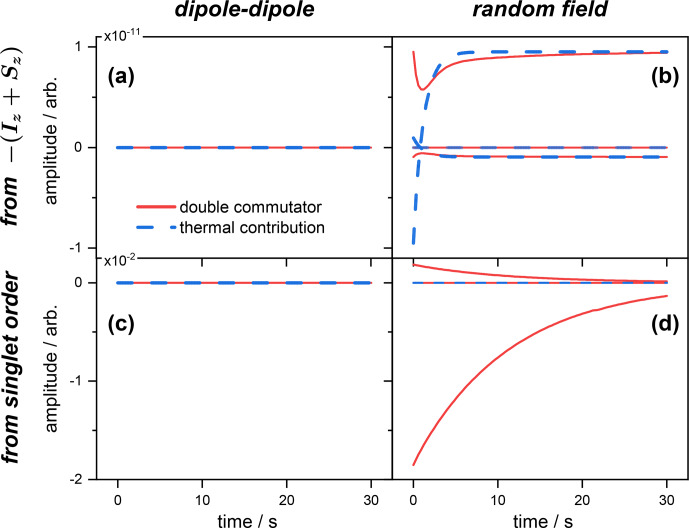
Same as Fig. [Fig Ch1.F1] for the expected value of the singlet operator $O=\frac{1}{4}1-I\cdot S$ .

Contributions from both relaxation mechanisms to the expected values in Eq. ([Disp-formula Ch1.E66]) were computed for the spins prepared either in a singlet state or inverted from thermal equilibrium. The individual terms entering the first and second sums on the right-hand side of Eq. ([Disp-formula Ch1.E66]) are depicted for the case of the magnetization $O=I_{z}+S_{z}$ (Fig. [Fig Ch1.F1]) and singlet state $O=\frac{1}{4}1-I\cdot S$ (Fig. [Fig Ch1.F2]) operators. The time evolutions of all the contributions to the rate of change of the expected value of the operator O(t) are depicted. When the spins are initially prepared in the state $-(I_{z}+S_{z})$ , the thermal contribution (blue curve) to the rate of change of the magnetization has no effect, and the only contribution to $\frac{\text{d}\langle O\rangle (t)}{\text{d}t}$ comes from the double commutator (red curve). This is the case for both relaxation mechanisms, i.e., dipolar and random field fluctuations (Fig. [Fig Ch1.F1]a and b). Moreover, the values chosen for the simulation imply that the dipolar contribution (of the double commutator in this case) to the total relaxation rate $\langle \dot{O}\rangle (t)$ is much larger than the one of the random field.

The situation is strikingly different when the spins are initially prepared in a singlet order. Here, the thermal correction (blue) is negligible with respect to the double commutator (red) contribution to the rate of change $\langle \dot{O}\rangle (t)$ for the dipole–dipole mechanism only (Fig. [Fig Ch1.F1]c). In contrast, for terms originating from the random field relaxation, both thermal and dipolar terms are of comparable orders of magnitude (Fig. [Fig Ch1.F1]c). These are the terms that cannot be neglected in an approximate solution of Eq. ([Disp-formula Ch1.E66]). Figure [Fig Ch1.F1]c also shows that the dipolar contribution to $\langle \dot{O}\rangle (t)$ increases with time, which is consistent with the progressive depletion of the singlet order (immune to dipolar relaxation). And, for random relaxation, which mainly affects the singlet order in this example, Fig. [Fig Ch1.F1]d illustrates the fact that the weight of the thermal contribution decays with time, with the concomitant increase in the double commutator term, which is also due to the progressive depletion of the singlet order.

The situation depicted in Fig. [Fig Ch1.F2] is different and shows the rate of change of the expected value 〈O〉(t) , where $O=\frac{1}{4}(I\cdot S)$ , for the same initial state conditions as above. In this case, the dipole–dipole simply does not contribute to $\langle \dot{O}\rangle (t)$ , as expected from symmetry considerations. This is, of course, the case whatever the initial state (inverted magnetization; see Fig. [Fig Ch1.F2]a) or singlet order (Fig. [Fig Ch1.F2]c). This illustrates the known fact that singlet state is immune to dipolar relaxation for symmetry reasons.

Alternatively, when the spins are prepared in the $-(I_{z}+S_{z})$ state, both thermal and double commutators contribute, albeit a negligible amount, showing that the spins evolve mostly towards magnetization (compare the scales with Fig. [Fig Ch1.F1]b) and that only a negligible part is transferred to a singlet order.

Interestingly, Fig. [Fig Ch1.F2]d shows that there is no thermal contribution (blue) to the rate $\langle \dot{O}\rangle (t)$ , and that, starting from a singlet order, its evolution can be predicted by discarding the thermal terms of Eq. ([Disp-formula Ch1.E66]) and, therefore, retaining the simple double commutator expression for the relaxation master equation.

#### 5.2.2 Singlet–triplet conversion

The recent achievement of the Lindblad approach was the description of the magnetization relaxation of a two-spin system prepared in a singlet state ([Bibr bib1.bibx5]). In that paper, detailed balance was enforced through the [Bibr bib1.bibx26] procedure, whereby spectral density functions are built from classical ones through the following transformation:

69 $$J(\omega {)}_{L,R}\to J^{\text{cl}}(\omega )e^{-\frac{\beta \omega }{2}},$$

where $J^{\text{cl}}(\omega )$ refers to the classical spectral density function. Equation ([Disp-formula Ch1.E69]) is one among several that have been proposed to make classical spectral density functions asymmetric so as to obey the detailed balance condition [Bibr bib1.bibx30]. In [Bibr bib1.bibx5], J(ω) was assumed to be any kind of spectral density function obtained through classical models, such as diffusion jumps, etc. The distinction between the left and right spectral densities that appear in the course of the conventional perturbative derivation of the master equation was not made there. Moreover, the detailed balance condition appears as an additional requirement, as this condition is not implied by Lindblad's approach that merely provides the general mathematical structure of the evolution equation obeyed by the density operator that complies with the requirements of quantum mechanics in the presence of a Markovian dissipative process [Bibr bib1.bibx18].

In the following, we derive the evolution of the magnetization of a two-spin system, using the singlet–triplet population basis, and compare the results obtained by both approaches. As above (and in [Bibr bib1.bibx5]) the relaxation super-operator $\hat{\Gamma }$ is the sum of contributions from mutual dipole–dipole relaxation ( ${\hat{\Gamma }}_{\text{DD}}$ ) and the interaction with a partially correlated random field ( ${\hat{\Gamma }}_{\text{ran}}$ ), as follows:

70 $$\hat{\Gamma }={\hat{\Gamma }}_{\text{DD}}+{\hat{\Gamma }}_{\text{ran}}.$$

The irreducible tensor operator $T_{\lambda \mu }$ representation is better suited to deriving analytical solutions for the problem at hand, where $\hat{\Gamma }$ is expressed, according to Eq. ([Disp-formula Ch1.E61]), as follows:

71 $$\begin{array}{rl} & {\hat{\Gamma }}_{\text{DD}}=\frac{6}{5}b_{12}^{2}\sum_{\mu =-2}^{\mu =2}J_{\text{DD}}^{\text{cl}}(\mu \omega^{0}){\hat{D}}^{\beta }(\mu \omega^{0})\left[T_{2\mu }^{(12)†},T_{2\mu }^{\left(12\right)}\right],\\ & {\hat{\Gamma }}_{\text{ran}}=\sum_{i,j=1}^{2}\kappa_{ij}\omega_{\text{rms}}^{\left(i\right)}\omega_{\text{rms}}^{\left(j\right)}\\ & \;\sum_{\mu =-1}^{\mu =1}J_{\text{ran}}^{\text{cl}}(\mu \omega^{0}){\hat{D}}^{\beta }(\mu \omega^{0})\left[T_{1\mu }^{(i)†},T_{1\mu }^{\left(j\right)}\right]. \end{array}$$

The $T_{\lambda \mu }$ are eigenoperators of the main Zeeman–Hamiltonian, and their expressions are shown in Appendix [App App1.Ch1.S3]. $\omega_{\text{rms}}^{\left(i\right)}$ is the root mean square fluctuation of the random field acting on spin $I_{i}$ , and in the isotropic case considered here, it is identical for both nuclei, so that $\omega_{\text{rms}}^{\left(1\right)}=\omega_{\text{rms}}^{\left(2\right)}$ . The coefficient $-1\leq \kappa_{12}\leq 1$ describes the degree of correlation of the random field fluctuations on the 1 and 2 nuclei. By definition, $\kappa_{11}=\kappa_{22}=1$ . In order to simplify the notations, we will henceforth drop the subscript ( $\kappa_{12}\to \kappa$ ).

In the extreme narrowing regime, where $\omega \tau_{\text{C, ran}}\ll 1$ , the spectral densities become frequency independent and J(ω)≈2τ . The dipole–dipole and random field contributions to the longitudinal relaxation rate constant $R_{1}=R_{1}^{\text{DD}}+R_{1}^{\text{ran}}$ are given by, according to Eq. ([Disp-formula Ch1.E61]), the following:

72 $$\begin{array}{rl} R_{1}^{\text{DD}} & =-\frac{\left(I_{z}|{\hat{\Gamma }}_{\text{DD}}|I_{z}\right)}{\left(I_{z}|I_{z}\right)}\\ & =\frac{3}{20}b_{12}^{2}\tau_{\text{C}}\left(4f_{\beta }(2\omega_{0})+4f_{\beta }(-2\omega_{0})\right.\\ & \left.+f_{\beta }(\omega_{0})+f_{\beta }(-\omega_{0})\right)\\ & =\frac{3}{2}b_{12}^{2}\tau_{\text{C}},\\ R_{1}^{\text{ran}} & =-\frac{\left(I_{z}|{\hat{\Gamma }}_{\text{ran}}|I_{z}\right)}{\left(I_{z}|I_{z}\right)}\\ & =\omega_{\text{rms}}^{2}\tau_{\text{ran}}\left(f_{\beta }(\omega_{0})+f_{\beta }(-\omega_{0})\right)\\ & =2\omega_{\text{rms}}^{2}\tau_{\text{ran}}, \end{array}$$

where $f_{\beta }(\omega )=\frac{2}{1+e^{\beta \omega }}$ . Equations ([Disp-formula Ch1.E72])–([Disp-formula Ch1.E75]) were obtained using the SpinDynamica software [Bibr bib1.bibx4]. It is interesting to note that, in this model, the relaxation rates do not depend on the temperature, which is in contrast to [Bibr bib1.bibx5]. This is not due to any approximation; rather, it arises from the fact that $f_{\beta }(\omega )+f_{\beta }(-\omega )=2$ , which is explicit in Eq. ([Disp-formula Ch1.E72]). Similarly, the singlet order relaxation rate is given by the following:

73 $$R_{\text{S}}=R_{\text{S}}^{\text{ran}}=-\frac{\left(I_{1}I_{2}|{\hat{\Gamma }}_{\text{ran}}|I_{1}I_{2}\right)}{\left(I_{1}I_{2}|I_{1}I_{2}\right)}=4\omega_{\text{rms}}^{2}\tau_{\text{ran}}(1-\kappa ),$$

which does not depend on the temperature. For sake of comparison with the results of [Bibr bib1.bibx5], we use the singlet–triplet population basis, where the singlet and triplet states are defined as follows:

74 $$\begin{array}{rl} & |S_{0}\rangle =(|\alpha_{1}\beta_{2}\rangle -|\beta_{1}\alpha_{2}\rangle )/\sqrt{2},\\ & |T_{+1}\rangle =|\alpha_{1}\alpha_{2}\rangle ,\\ & |T_{0}\rangle =(|\alpha_{1}\beta_{2}\rangle +|\beta_{1}\alpha_{2}\rangle )/\sqrt{2},\\ & |T_{-1}\rangle =|\beta_{1}\beta_{2}\rangle , \end{array}$$

where |α〉 and |β〉 denote the Zeeman spin states of an isolated spin 1/2 (one-half) nucleus with z projection of +1/2 and -1/2 . In this representation, the population block of relaxation super-operator Eq. ([Disp-formula Ch1.E71]) is given by Eq. (76).


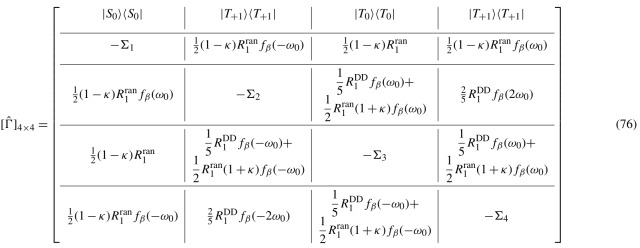


In Eq. (76), $\Sigma_{i}$ denotes the sum of the terms alongside respective column. This matrix is very similar to one introduced in [Bibr bib1.bibx5] but with the substitution of the term θ(ω)=exp(-βω/2) by $f_{\beta }(\omega )$ .

In the high temperature limit, both terms become approximately equal, $\theta (\omega )\approx f_{\beta }(\omega )\approx 1-\frac{\beta \omega }{2}$ , and the difference between θ(ω) and $f_{\beta }(\omega )$ becomes significant only in the case of extremely low temperatures or very high frequencies. As could be expected in this limit, the time evolution of the z magnetization is given by the same bi-exponential behavior as in [Bibr bib1.bibx5], as follows:

77 $$\langle I_{z}(t)\rangle /\langle I_{z}^{\mathrm{eq}}\rangle \approx 1+A_{1}e^{-R_{1}t}+A_{\text{S}}e^{-R_{\text{S}}t},$$

with the same coefficients $A_{1}=\frac{R_{\text{S}}}{2(R_{1}-R_{\text{S}})}$ and $A_{\text{S}}=\frac{-2R_{1}+R_{\text{S}}}{2(R_{1}-R_{\text{S}})}$ . The fact that $A_{1}$ and $A_{\text{S}}$ are the ones found in [Bibr bib1.bibx5] is expected because, in this limit, $R_{1}$ and $R_{\text{S}}$ do not depend on the temperature factor. In the usual conditions of high but not infinite temperature, it is found that the next nonzero terms in the expansions of $A_{1}(\beta )$ and $A_{\text{S}}(\beta )$ are of the degree $\beta^{2}$ and, therefore, do not contribute in the regime where ωβ≪1 . These expressions can be found in Appendix [App App1.Ch1.S4].

The foregoing discussion has shown that, in the high temperature approximation, the exact thermalization procedure of the spectral density function is irrelevant, as all models are equivalent in these conditions. Indeed, in a field of 23.5 T ( 1000 MHz resonance proton frequency), the temperature at which $\hbar \omega_{H}\approx kT$ , where both approaches may lead to significant differences, is T≈50 mK. These are unrealistic experimental conditions. Alternatively, the master equation of Eqs. ([Disp-formula Ch1.E58])–([Disp-formula Ch1.E61]) may well be of use in the context of dynamic nuclear polarization (DNP) to describe the electron spin–lattice relaxation outside of the high temperature limit through direct spin–phonon coupling at temperatures below 4 K and at high fields, where this process is predominant.

## 6 Conclusion: a remark on semi-classical theory

In the semi-classical viewpoint (as in [Bibr bib1.bibx1], for instance), the effect of the bath is taken into account through a stochastic spin Hamiltonian, the spatial part of which is a function of the lattice variables and is a random function of time. It is usually understood that this approach does not comply with the Boltzmann equilibrium of the bath. Besides, the stationary state reached by the spin density operator is left undetermined by the master equation so that it must be enforced by the supplementary ad hoc assumption that the spins return to the Boltzmann distribution of the spin populations. A recent analysis by [Bibr bib1.bibx5] showed that the usual semi-classical master equation was not able to predict the correct magnetization evolution of a two-spin system prepared in a singlet state.

Thus, the usual semi-classical inhomogeneous master equation provides erroneous predictions in this case. The latter is obtained when the thermal corrections to the double commutator part of the relaxation operator are retained to the first order in the largest eigenvalue $\frac{\omega_{p}^{q}}{kT}$ , and the relaxation operator reduces to a double commutator (low-order case). However, the equilibrium density operator is not a stationary solution in this case, and therefore, a correction term is added to the master equation, leading to the same result as the usual semi-classical master equation ([Bibr bib1.bibx14]). And so, when the low-order assumption is not verified, as in the case of a spin system prepared in the singlet state, this description becomes inconsistent.

As shown above, the non-commutation of the bath operators has critical consequences, leading to the lattice-temperature-dependent terms in the master equation, and it is only when the bath operators $[B^{-q}(t),B^{q}(0)]=0$ that one recovers the double commutator expression, with the additional property $J_{\text{L}}(\omega )=J_{\text{R}}(\omega )$ , so that the Kubo relation imposes an infinite lattice temperature. This illustrates how the finite temperature of the lattice is conveyed to the spins through non-commutation of the bath operators of the coupling Hamiltonian.

The conventional semi-classical approach, where spin–bath interactions are represented by random spin Hamiltonians, has the following two simultaneous consequences: the structure of the relaxation operator is affected in such a way that the master equation takes the form of a double commutator, and since $J_{\text{L}}(\omega )=J_{\text{R}}(\omega )$ , the system cannot evolve to a thermodynamic equilibrium associated with a finite temperature. In this case, the detailed balance property is conserved but only in the special case of infinite lattice temperature. In fact, since the detailed balance is statistical by nature, it is per se compatible with a semi-classical approach. If, on the other hand, a detailed balance is taken into account in the semi-classical theory of NMR relaxation, so that $J(-\omega )=e^{-\hbar \omega /kT}J(\omega )$ and the general relations Eq. ([Disp-formula Ch1.E48]) or ([Disp-formula Ch1.E50]) obeyed by correlation functions are retained, then it is easy to show from the symmetry properties of the spectral density function that the semi-classical Redfield equation ([Bibr bib1.bibx1]) in the following:

78 $${\dot{\sigma }}_{\text{S}}^{*}(t)=-\frac{1}{2}\sum_{q,p}J(\omega_{p}^{q})\left[S_{p}^{-q},\left[S_{p}^{q},\sigma (t)\right]\right],$$

is obeyed with this definition of J(ω) . However, the expected equilibrium density operator is not a stationary state of Eq. ([Disp-formula Ch1.E76]) in this case, which illustrates the (also known) fact that this condition alone is insufficient to completely determine the transition probabilities of the bath in the absence of a dynamical model for the latter. On the other hand, it is possible to describe the dynamics of a classical system where microscopic irreversibility, i.e., detailed balance, is ensured. This is straightforward from the definition of the correlation function of a phase variable in classical mechanics, $\langle A(t)B^{*}\rangle =\int \text{d}q\text{d}p\rho^{e}B^{*}e^{iLt}A$ , where L is the classical Liouvillian acting on the phase space ([Bibr bib1.bibx11]). In addition, general procedures have been used that provide Fokker–Planck or master equations for diffusion that obey the detailed balance condition, yielding classical spectral density functions that comply with the Boltzmann equilibrium distribution and the classical laws of motion of the bath see, for instance,, in particular in the context of magnetic resonance ([Bibr bib1.bibx27]). As stated in several instances in this work, in the fully quantum approach, detailed balance is ensured by assuming that the bath is in a stationary state defined by a Boltzmann distribution of its energy states. Thus, the irreducible difference between the semi-classical and the fully quantized theory lies in the fact that the bath operators do not mutually commute, which prevents the expression in Eq. ([Disp-formula Ch1.E26]) from reducing to the double commutator. Both thermodynamic and quantum mechanical effects are thus entangled in the fully quantum mechanical treatment of relaxation.

## Data Availability

No data sets were used in this article.

## Appendix A Derivation of Eq. (19)

A1ddtσ(t)=LSσ(t)+eLSt∫0tdt′trBL1∗(t)L1∗(t′)ρBeσ∗(t)A2=LSσ(t)+∫0tdt′trBeLStL1∗(t)L1∗(t′)ρBee-tLSσ(t)A3=LSσ(t)+∫0tdt′trBeLSte-(LS+LB)tL1e(LS+LB)te-(LS+LB)t′L1e(LS+LB)t′e-tLSρBeσ(t)A4=LSσ(t)+∫0tdt′trBe-LBtL1e(LS+LB)te-(LS+LB)t′L1e(LS+LB)t′e-tLSρBeσ(t)A5=LSσ(t)+∫0tdt′trBL1e(LS+LB)te-(LS+LB)t′L1e(LS+LB)t′e-tLSe-LBtρBeσ(t)A6=LSσ(t)+∫0tdt′trBL1e-(LS+LB)(t′-t)L1e(LS+LB)(t′-t)ρBeσ(t).

​​​​​​​In this derivation, the invariance of the trace to the circular permutations has been used, together with the fact that $[L_{\text{B}},\rho_{\text{B}}^{e}]=0$ , since the bath is in a stationary state.

## Appendix B Evaluation of the terms of Eq. (44)

The correlation functions involved in Eq. ([Disp-formula Ch1.E43]) are as follows:

B1
$$\begin{array}{rl} & \frac{1}{2}J_{\text{R}}^{q,-q}(\omega_{p}^{q})\\ & =\int_{0}^{\infty }\text{d}\tau \langle B^{q}B^{-q}(\tau )\rangle e^{-i\omega_{p}^{q}\tau }\\ & =\int_{0}^{\infty }\text{d}\tau \frac{1}{L}\sum_{f,f^{\prime }}\langle f|B^{q}|f^{\prime }\rangle \langle f^{\prime }|B^{-q}|f\rangle \\ & \;e^{i(f^{\prime }-f)\tau }e^{-\beta f}e^{-i\omega_{p}^{q}\tau }\\ & =\int_{0}^{\infty }\text{d}\tau \frac{1}{L}\sum_{f,f^{\prime }}|\langle f|B^{q}|f^{\prime }\rangle {|}^{2}e^{i(f^{\prime }-f)\tau }e^{-\beta f}e^{-i\omega_{p}^{q}\tau }\\ & \approx \frac{1}{2}\frac{1}{L}\sum_{f,f^{\prime }}|\langle f|B^{q}|f^{\prime }\rangle {|}^{2}e^{-\beta f}\int_{-\infty }^{\infty }e^{i(f^{\prime }-f-\omega_{p}^{q})\tau }\text{d}\tau \\ & =\frac{1}{2}\frac{1}{L}\sum_{f,f^{\prime }}|\langle f|B^{q}|f^{\prime }\rangle {|}^{2}e^{-\beta f}\delta (f^{\prime }-f-\omega_{p}^{q})\\ & =\frac{1}{2}\frac{1}{L}\sum_{f}|\langle f|B^{q}|f+\omega_{p}^{q}\rangle {|}^{2}e^{-\beta f}, \end{array}$$

since $B^{-q}=B^{q†}$ . Similarly, one has the following:

B2
$$\begin{array}{rl} & \frac{1}{2}J_{\text{L}}^{-q,q}(\omega_{p}^{q})\\ & =\int_{0}^{\infty }\text{d}\tau \langle B^{-q}(\tau )B^{q}\rangle e^{-i\omega^{q}\tau }\\ & =\frac{1}{L}\sum_{f,f^{\prime }}\langle f|B^{-q}|f^{\prime }\rangle \langle f^{\prime }|B^{q}|f\rangle e^{-\beta f}\\ & \;\int_{0}^{\infty }\text{d}\tau e^{i(f-f^{\prime })\tau }e^{-i\omega^{q}\tau }\\ & =\frac{1}{2}\frac{1}{L}\sum_{f,f^{\prime }}|\langle f|B^{-q}|f^{\prime }\rangle {|}^{2}e^{-\beta f}\int_{-\infty }^{\infty }e^{i(f-f^{\prime }-\omega^{q})\tau }\\ & =\frac{1}{2}\frac{1}{L}\sum_{f,f^{\prime }}|\langle f|B^{-q}|f^{\prime }\rangle {|}^{2}e^{-\beta f}\delta (f-f^{\prime }-\omega^{q}). \end{array}$$

Noting that $\left|\langle f|B^{-q}|f^{\prime }\rangle |=|B_{ff^{\prime }}^{-q}|=|B_{ff^{\prime }}^{q†}|=|B_{f^{\prime }f}^{q*}|=|B_{f^{\prime }f}^{q}\right|$ , and exchanging indices $f↔f^{\prime }$ , one obtains, from Eq. ([Disp-formula App1.Ch1.S2.E84]) the following:

B3
$$\begin{array}{rl} & \frac{1}{L}\sum_{f,f^{\prime }}|\langle f|B^{-q}|f^{\prime }\rangle {|}^{2}e^{-\beta f}\delta \left(f-f^{\prime }-\omega_{p}^{q}\right)\\ & =\frac{1}{L}\sum_{f,f^{\prime }}|\langle f^{\prime }|B^{q}|f\rangle {|}^{2}e^{-\beta f}\delta \left(f-f^{\prime }-\omega_{p}^{q}\right)\\ & =\frac{1}{L}\sum_{f,f^{\prime }}|\langle f|B^{q}|f^{\prime }\rangle {|}^{2}e^{-\beta f^{\prime }}\delta \left(f^{\prime }-f-\omega_{p}^{q}\right)\\ & =\frac{1}{L}\sum_{f}|\langle f|B^{q}|f+\omega_{p}^{q}\rangle {|}^{2}e^{-\beta \left(f+\omega_{p}^{q}\right)}\\ & =e^{-\beta \omega_{p}^{q}}\frac{1}{L}\sum_{f}|\langle f|B^{q}|f+\omega_{p}^{q}\rangle {|}^{2}e^{-\beta f}\\ & =e^{-\beta \omega^{q}}J^{\text{R}}\left(\omega_{p}^{q}\right). \end{array}$$

Besides, it immediately follows from the definitions of $J_{\text{R}}^{q}(\omega )$ and $J_{\text{L}}^{q}(\omega )$ so that, in the following:

B4
$$J_{\text{R}}^{q}(-\omega )=J_{\text{R}}^{-q}(\omega ).$$

## Appendix C Eigenoperators for a homonuclear coupled spin 1/2 pair

In the case of a homonuclear spin pair, the main Hamiltonian is defined as follows:

C1
$$H_{Z}=\omega^{0}\left(I_{1z}+I_{2z}\right),$$

where $\omega^{0}=-\gamma B$ , γ is the magnetogyric ratio, and B is the field strength. The eigenoperators for this Hamiltonian are summarized in Table [Table App1.Ch1.S3.T1]. The eigenoperators are denoted by $T_{\lambda \mu }^{\left(ij\right)}$ . The superscript (ij) indicates the angular momentum coupling of spins $I_{i}$ and $I_{j}$ , resulting in a spherical tensor operator of the total angular momentum λ and z -angular momentum μ .

**Table C1 App1.Ch1.S3.T1:** Eigenoperators of Hamiltonian for a homonuclear coupled spin 1/2 pair.

μ\λ	2	1
± 2	$\frac{1}{2}I_{1}^{\pm }I_{2}^{\pm }$	–
± 1	$\mp \left(I_{1}^{\pm }I_{2}^{z}+I_{1z}I_{2}^{\pm }\right)$	$\mp \frac{1}{\sqrt{2}}I_{j}^{\pm }$
0	$-\frac{1}{2\sqrt{6}}\left(I_{1}^{+}I_{2}^{-}+I_{1}^{-}I_{2}^{+}-4I_{1z}I_{2z}\right)$	$I_{jz}$

## Appendix D Expansion coefficient for the magnetization evolution solution

The coefficients from the Eq. ([Disp-formula Ch1.E75]) are in fact temperature dependent, as follows:

D1
$$\begin{array}{rl} & A_{1}(\beta )=A_{1}+C_{1}\beta^{2}+O\left(\beta^{4}\right)\\ & A_{\text{S}}(\beta )=A_{\text{S}}+C_{\text{S}}\beta^{2}+O\left(\beta^{4}\right), \end{array}$$

where the coefficients $A_{1}$ and $A_{\text{S}}$ were shown before in the main text, and the coefficients $C_{1}$ and $C_{\text{S}}$ are defined as follows:

D2
$$\begin{array}{rl} & C_{1}=\\ & \omega_{0}^{2}\frac{\begin{array}{c} R_{\text{S}}\left(600R_{1}^{3}-20R_{1}^{2}\left(4416R_{1}^{\text{dd}}+35R_{\text{S}}\right)\right.\\ \left.-5R_{\text{S}}\left(17\;280{\left(R_{1}^{\text{dd}}\right)}^{2}+2958R_{1}^{\text{dd}}R_{\text{S}}+5R_{\text{S}}^{2}\right)\right.\\ \left.+2R_{1}\left(43\;488{\left(R_{1}^{\text{dd}}\right)}^{2}+51\;120R_{1}^{\text{dd}}R_{\text{S}}+125R_{\text{S}}^{2}\right)\right) \end{array}}{5760R_{1}\left(150R_{1}-149R_{1}^{\text{dd}}-25R_{\text{S}}\right){\left(R_{1}-R_{\text{S}}\right)}^{2}}\\ & C_{\text{S}}=-C_{1}. \end{array}$$

## Code availability

The codes used in the simulations can be found at https://gitlab.com/phagost/linblad_simulations (Rodin, 2022).

## References

[bib1.bibx1] Abragam A (1961). Principles of Nuclear Magnetism.

[bib1.bibx2] Alicki R, Lendi K (2007). Quantum Dynamical Semigroups and Applications.

[bib1.bibx3] Barbara TM (2021). The Lindbladian form and the reincarnation of Felix Bloch's generalized theory of relaxation. Magn Reson.

[bib1.bibx4] Bengs C, Levitt MH (2018). SpinDynamica: Symbolic and numerical magnetic resonance in a Mathematica environment. Magn Reson Chem.

[bib1.bibx5] Bengs C, Levitt MH (2020). A master equation for spin systems far from equilibrium. J Magn Reson.

[bib1.bibx6] Bloch F (1956). Dynamical Theory of Nuclear Induction. II. Phys Rev.

[bib1.bibx7] Bloch F (1957). Generalized Theory of Relaxation. Phys Rev.

[bib1.bibx8] Bloembergen N, Purcell EM, Pound RV (1948). Relaxation Effects in Nuclear Magnetic Resonance Absorption. Phys Rev.

[bib1.bibx9] Egorov S, Skinner J (1998). Semiclassical approximations to quantum time correlation functions. Chem Phys Lett.

[bib1.bibx10] Egorov SA, Everitt KF, Skinner JL (1999). Quantum Dynamics and Vibrational Relaxation. J Phys Chem A.

[bib1.bibx11] Evans DJ, Moriss G (2008). Statistical Mechanics Of Nonequilibrium Liquids.

[bib1.bibx12] Frommhold L (1993). Collision-induced absorption in gases, Cambridge monographs on atomic, molecular, and chemical physics.

[bib1.bibx13] Goldman M (2001). Formal Theory of Spin-Lattice Relaxation. J Magn Reson.

[bib1.bibx14] Hubbard PS (1961). Quantum-Mechanical and Semiclassical Forms of the Density Operator Theory of Relaxation. Rev Mod Phys.

[bib1.bibx15] Jeener J (1982). Superoperators in Magnetic Resonance. Adv Magn Reson.

[bib1.bibx16] Kubo R (1957). Statistical-Mechanical Theory of Irreversible Processes. I. General Theory and Simple Applications to Magnetic and Conduction Problems. J Phys Soc Jpn.

[bib1.bibx17] Levitt M, Bari LD (1992). Steady state in magnetic resonance pulse experiments. Phys Rev Lett.

[bib1.bibx18] Lindblad G (1976). On the Generators of Quantum Dynamical Semigroups. Commun Math Phys.

[bib1.bibx19] Maimbourg T, Basko DM, Holzmann M, Rosso A (2021). Bath-Induced Zeno Localization in Driven Many-Body Quantum Systems. Phys Rev Lett.

[bib1.bibx20] Nathan F, Rudner MS (2020). Universal Lindblad equation for open quantum systems. Phys Rev B.

[bib1.bibx21] Ramirez R, López-Ciudad T (2004). Quantum corrections to classical time-correlation functions: Hydrogen bonding and anharmonic floppy modes. J Chem Phys.

[bib1.bibx22] Redfield AG (1957). On the theory of relaxation processes. IBM J Res & Dev.

[bib1.bibx23] Redfield AG (1965). The Theory of Relaxation Processes. Adv Magn Reson.

[bib1.bibx24] Risken H (1972). Solutions of the Fokker-Planck equation in detailed balance. Zeit Phys.

[bib1.bibx25] Rodin B Gitlab.

[bib1.bibx26] Schofield P (1960). Space-time correlation function formalism for slow neutron scattering. Phys Rev Lett.

[bib1.bibx27] Stillman AE, Freed JH (1980). Stochastic modeling of generalized Fokker–Planck equations. I.. J Chem Phys.

[bib1.bibx28] VanKampen N (1981). Stochastic Processes in Physics and Chemistry.

[bib1.bibx29] Wassam WA, Balderas-Lopez JA, Torres-Vega G (1980). Stationarity of time correlation functions for globally linear classical systems and its consequences. J Math Phys.

[bib1.bibx30] White J, Velasco S, Hernandez AC, Luis D (1988). On the quantum time autocorrelation function. Phys Lett A.

